# *Sleeve gastrectomy* dans la prise en charge chirurgicale de l´obésité au Centre Hospitalier d'Essos (Yaoundé, Cameroun): évaluation rétrospective de la perte pondérale à moyen terme sur une série de cas

**DOI:** 10.11604/pamj.2024.49.49.32728

**Published:** 2024-10-22

**Authors:** Guy Aristide Bang, Blondel Nana Oumarou, Eric Patrick Savom, Johanna Joyce Mbianda Nketcha, Arthur Essomba

**Affiliations:** 1 Service de Chirurgie Viscérale et Laparoscopique, Département de Chirurgie et Spécialités, Faculté de Médecine et des Sciences Biomédicales de l'Université de Yaoundé I, Yaoundé, Cameroun,; 2 Département de Chirurgie et Spécialités, Faculté de Médecine et des Sciences Biomédicales de l'Université de Yaoundé I, Yaoundé, Cameroun,; 3 Service de Chirurgie, Hôpital Général de Yaoundé, Yaoundé, Cameroun

**Keywords:** Obésité, chirurgie bariatrique, *sleeve gastrectomy*, perte pondérale, Cameroun, obesity, bariatric surgery, sleeve gastrectomy, weight loss, Cameroon

## Abstract

Si la prévalence du surpoids et de l'obésité augmente en Afrique, la pratique de la chirurgie bariatrique demeure marginale dans notre pays le Cameroun. Les résultats sur la perte pondérale après « sleeve gastrectomy » (SG), technique la plus usitée de chirurgie bariatrique dans le monde, n'ont pas fait l'objet d'une étude à ce jour dans notre contexte. Les dossiers de tous les patients ayant eu une SG dans notre service de chirurgie entre le 1^er^ janvier 2016 et le 30 septembre 2020 ont été rétrospectivement consultés. La perte pondérale à moyen terme, un an après la chirurgie, était le principal résultat investigué. Cette perte pondérale était calculée à l'aide de l'indice de masse corporelle (IMC) postopératoire et du pourcentage de perte de l'excès de poids (%PEP). Les critères de Reinhold ont été utilisés pour évaluer le %PEP. Nous avons colligé 21 patients parmi lesquels 19 (90,5%) était de sexe féminin. L'âge moyen des patients était de 40,3±10,8 ans avec un IMC moyen de 44,9±7,4 kg/m^2^. Tous les patients avaient au moins une comorbidité liée à l'obésité. Toutes les procédures ont été conduites par voie coelioscopique, sans conversion, avec une durée moyenne de 192,2±52,8 min. Un an après la chirurgie, l'IMC moyen de nos patients était de 32,51± 3,7 kg/m^2^ et le %PEP moyen de 63,35±6,5%. Selon les critères de Reinhold, le %PEP était considéré comme satisfaisant. Dans notre contexte, la SG donne des résultats satisfaisants sur la perte pondérale à moyen terme.

## Abstract (en)

While the prevalence of overweight and obesity is rising in Africa, the practice of bariatric surgery remains limited in our country, Cameroon. Weight loss outcomes following sleeve gastrectomy (SG), the most widely used bariatric surgery technique worldwide, have not yet been studied in our context. The medical records of all patients who underwent SG in our surgery department between January 1, 2016, and September 30, 2020, were reviewed retrospectively. The primary outcome studied was medium-term weight loss, one year after surgery. Weight loss was calculated using the postoperative body mass index (BMI) and the percentage of excess weight loss (%EWL). Reinhold criteria were used to assess the %EWL. We collected data from the medical records of 21 patients, of whom 19 (90.5%) were female. The average age of patients was 40.3±10.8 years, with a mean BMI of 44.9±7.4 kg/m^2^. All patients had at least one obesity-related comorbidity. All procedures were performed laparoscopically, without conversion, with a mean duration of 192.2±52.8 min. One year after surgery, the mean BMI was 32.51±3.7 kg/m^2^ and the mean %EWL was 63.35±6.5%. According to Reinhold’s criteria, the %EWL was considered satisfactory. In our context, SG provides satisfactory results for medium-term weight loss.

## Introduction

Avec l'occidentalisation des modes de vie, l'obésité est devenue un problème de santé publique dans les pays en voie de développement. En Afrique noire, la prévalence de l'obésité a été estimée à 34% [1] et à 27,2% en Afrique du Sud [2]. Notre pays, le Cameroun, n'est pas épargné; on estime à 15,1% la prévalence de l'obésité dans la population globale et à 12,5% chez les enfants âgés de 3 à 13 ans [3,4]. L'obésité dans notre contexte est associée à de nombreuses comorbidités [3-6].

La chirurgie bariatrique ou chirurgie de l'obésité est indiquée chez les patients âgés de 18 à 60 ans avec un indice de masse corporelle (IMC) ≥40,0 kg/m^2^ ou avec un IMC compris entre 35 et 39,9 kg/m^2^ et associé à des comorbidités [7]. Si plusieurs techniques de chirurgie bariatrique existent, la *sleeve gastrectomy* (SG) ou gastrectomie longitudinale demeure la plus usitée dans le monde [8,9]. Cette technique consiste en la résection de la grande courbure de l'estomac pour ne laisser en place qu'un manchon gastrique semblable, en forme et en taille, à une banane [10]. La «popularité» de cette technique réside dans le fait qu'elle ne nécessite pas la réalisation d'une anastomose ou d'une dérivation bilio-digestive, réduisant ainsi la morbidité postopératoire des patients [11]. La SG entraine une perte de 54,9 à 70% de l'excès de poids dans les 12 mois suivants sa réalisation [12,13].

La pratique de la SG demeure récente et peu courante au Cameroun. Peu d'études existent à ce jour sur les résultats de cette technique dans notre contexte. En 2016, la SG a commencé à être réalisée dans notre service; le but de ce travail était d'en évaluer les résultats sur la perte pondérale à moyen terme (1 an après l'intervention chirurgicale).

## Méthodes

**Type et cadre de l'étude:** il s'agissait d'une série de cas avec recueil rétrospectif de données. Elle a eu pour cadre le service de chirurgie viscérale et laparoscopie du Centre Hospitalier d'Essos. Il s'agit d'une structure sanitaire parapublique de référence de la ville de Yaoundé, la capitale du Cameroun. L'activité de coelio-chirurgie a débuté dans ce service en 2010 et celle de chirurgie bariatrique par SG depuis 2016 par la même équipe.

**Figure 1:**
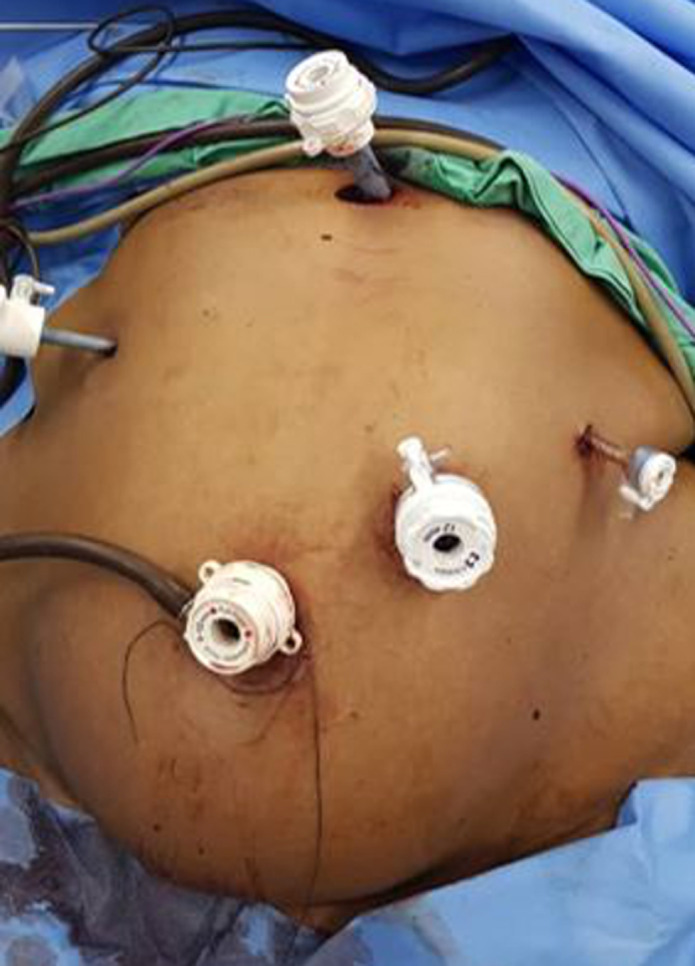
position des trocarts pour la réalisation d'une procédure de sleeve gastrectomy

**Figure 2:**
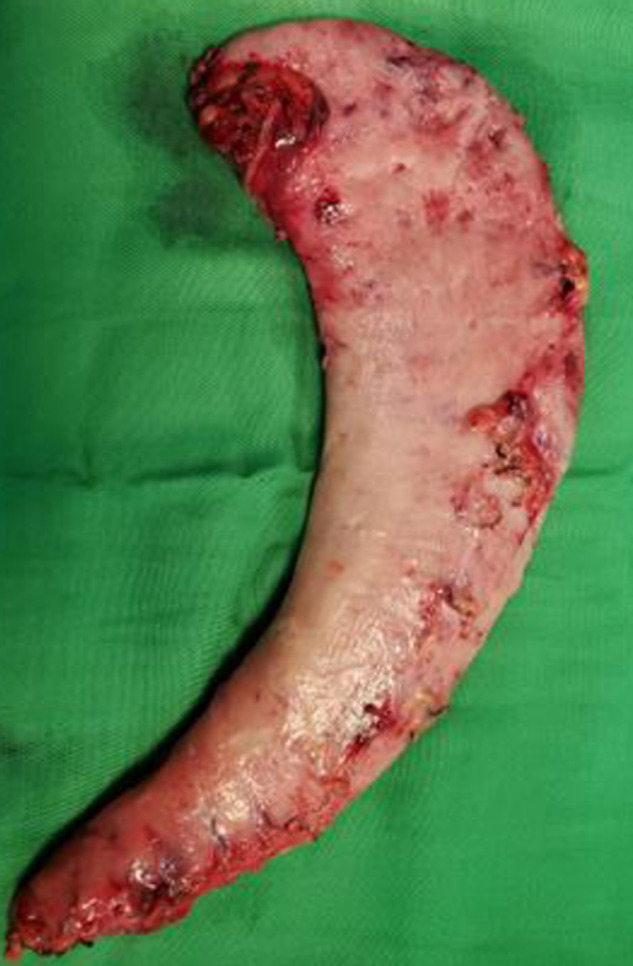
pièce opératoire d'une sleeve gastrectomy présentant les 2/3 de l'estomac emportés

**Population d'étude:** rétrospectivement, nous avons colligé les dossiers complets de patients ayant eu une SG entre le 1^er^ janvier 2016 et le 30 septembre 2020. Ainsi, nous pouvions avoir au moins un an de recul de suivi sur tout patient opéré. Les patients ayant eu une chirurgie bariatrique par une autre technique que la SG n'a pas été inclus dans cette étude. La décision de réaliser une SG était prise en réunion pluridisciplinaire et après que le patient ait été informé des bénéfices, des risques et du calendrier de suivi postopératoire. Tous nos patients étaient opérés sous anesthésie générale avec intubation orotrachéale. L'installation du patient se faisait selon la position française: décubitus dorsal avec un proclive de 30 à 45°, membres inférieurs écartés, bras en croix. L'opérateur principal était entre les jambes du patient, le premier aide à sa droite et l'instrumentiste à sa gauche. Le premier trocart (10 mm) était placé en sus-ombilical en «open-coelioscopy» et le pneumopéritoine réalisé via cet abord. Quatre autres trocarts étaient introduits dans la cavité péritonéale sous le contrôle de la vue (Figure 1) dont 2 de 10 mm (pararectal droit et épigastrique) et deux de 5 mm (sous costal droit et gauche). Un décollement gastro-épiploïque à l'aide d'une pince bipolaire type thermofusion, était par la suite réalisé avec une mobilisation complète de la grande courbure gastrique. Les ligaments gastro-pancréatiques postérieurs étaient sectionnés. L'anesthésiste introduisait par la suite une sonde de Faucher de 36-Fr dans l'estomac, permettant ainsi son calibrage. La gastrectomie longitudinale était alors réalisée à l'aide d'une pince endo-GIA, en débutant à 4 cm du pylore et s'étendant jusqu'à l'angle de Treitz. Trois à cinq recharges étaient utilisées pour cette section gastrique. La pièce opératoire (Figure 2) était ressortie de l'abdomen via l'abord sus-ombilical et un surjet sur la tranche de section réalisé à l'aide d'un fil résorbable auto-bloquant (V-Loc®). Les patients étaient revus systématiquement 1 mois, 3 mois, 6 mois et 1 an après l'intervention chirurgicale. Durant ces rendez-vous de contrôle, la prise pondérale se faisait sur le pèse-personne du service par une infirmière formée à cet effet et sous le contrôle d'un des membres de l'équipe chirurgicale. L'évolution pondérale était consignée sur une courbe dans le dossier du patient.

**Collecte de données:** la consultation des registres de comptes rendus opératoires nous a permis d'identifier les patients ayant eu une SG durant la période d'étude. Les dossiers de ceux-ci ont été par la suite consultés, et ceux qui remplissaient nos critères d'inclusion ont été colligés. Une fiche de collecte de données était renseignée avec comme variables d'études: l'âge, le sexe, l'IMC préopératoire, l'excès de poids préopératoire, les comorbidités, la durée de l'intervention chirurgicale, la conversion et la perte pondérale un an après la chirurgie. La perte pondérale était le principal résultat investigué. La perte pondérale était évaluée 1 mois, 3 mois, 6 mois et 12 mois après la chirurgie à l'aide de l'IMC postopératoire et du pourcentage de perte de l'excès de poids (%PEP). Le %PEP était calculé par les deux premiers investigateurs.

**Définitions:** les dossiers de patients étaient considérés comme complets s'ils contenaient toutes les variables d'étude. Le moyen terme a été défini comme l'évaluation un an après l'intervention chirurgicale. Le %PEP était calculé selon la formule suivante: (Poids initial-Poids actuel)/(Poids initial) x100.

**Analyse statistique:** toutes ces données ont été transformées en variables et analysées à l'aide du logiciel SPSS 12.0. Les données quantitatives ont été exprimées par leur moyenne ± écart-type et les données qualitatives par leurs fréquences. L'efficacité de la chirurgie bariatrique sur la perte pondérale a été évaluée en fonction du %PEP en accord avec les critères de Reinhold [14]: elle était considérée comme excellente si le %PEP était supérieur à 75%, satisfaisante pour un %PEP situé entre 50 et 75%, modérée entre 25 et 50% et comme un échec pour un %PEP inférieur à 25%.

**Considérations éthiques:** nous avons obtenu l'accord du comité d'éthique de la Faculté de Médecine et des Sciences Biomédicales de l'université de Yaoundé I avant le début de l'étude (Numéro 0927/UY1/FMSB/VDRC/DAASR/CSD), ainsi que l'autorisation de recherche de la direction du Centre Hospitalier d'Essos. Le respect de la confidentialité des informations recueillies a été scrupuleusement observé et les images opératoires anonymées.

## Résultats

**Caractéristiques générales de la population d'étude (Tableau 1):** durant la période d'étude, 21 patients ont eu une SG dans notre service et tous ont été colligés dans notre travail. La majorité d'entre-eux (19, soit 90,5%) étaient de sexe féminin. L'âge moyen des patients était de 40,3±10,8 ans. L'IMC moyen de 44,9±7,4 kg/m^2^.

**Tableau 1:** caractéristiques de la population d'étude

Données	Effectif/valeur	Pourcentage (%)
**Sexe**		
Masculin	2	9,5
Féminin	19	90,5
Age moyen	40,3±10,8 ans	-
IMC moyen	44,9±7,4 kg/m^2^	-
Excès de poids moyen	64,5±20,3kgs	
**Comorbidité liée à l'obésité**		
Ostéoarhtrite	21	100
Apnée du sommeil	8	38,1
Hypertension artérielle	4	19,1
Diabète	3	14,3
Dyslipidémie	3	14,3
Dépression	1	4,7
Goutte	1	4,7

**Comorbidités liées à l'obésité (Tableau 1):** tous ces patients présentaient au moins une comorbidité parmi lesquels les plus fréquentes étaient: une ostéo-arthrite dans 21 cas (100%), une apnée du sommeil dans 8 cas (38,1%), une hypertension artérielle dans 4 cas (19,1%).

**Intervention chirurgicale:** toutes les procédures ont été conduites par voie coelioscopique avec une durée moyenne de 192,2±52,8 min et aucune conversion n'a été réalisée. La durée moyenne d'hospitalisation était de 4,7±1,1 jours.

**Figure 3:**
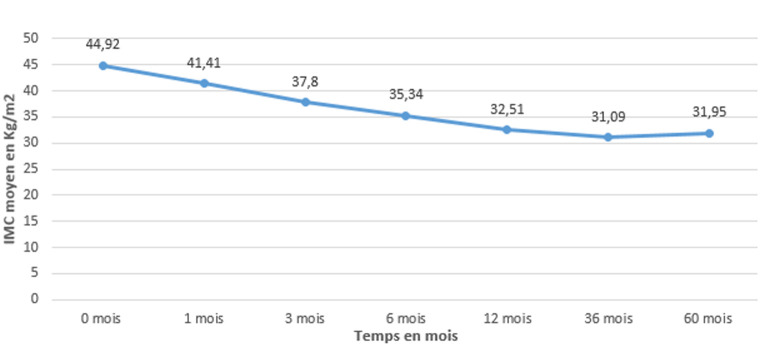
évolution postopératoire de l'indice de masse corporelle moyen des patients après sleeve gastrectomy

**Figure 4:**
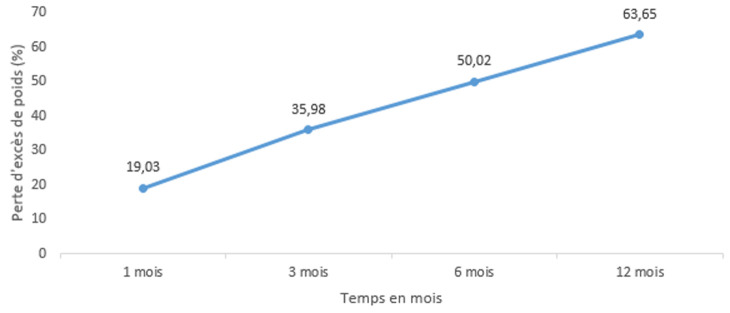
évolution postopératoire du pourcentage de perte de l'excès de poids après *sleeve gastrectomy*

**Evaluation de la perte pondérale après chirurgie:** en postopératoire, l'indice de masse corporelle moyen de nos patients était passé de 44,9±7,4 kg/m^2^ à 35,34±5,2 kg/m^2^ à 6 mois et à 32,51±3,7 kg/m^2^ à 1 an. Passé ce délai, on notait une stagnation de l'IMC. La Figure 3 présente l'évolution de l'IMC des patients en postopératoire. Le %PEP moyen en postopératoire (Figure 4) est passé de 19,03±2,1% à un mois à 63,35±6,5% à un an.

## Discussion

Le but de cette étude était d'évaluer la perte pondérale à moyen terme (1 an) chez les patients ayant eu une chirurgie bariatrique par SG dans notre service. L'IMC moyen de nos patients était passé de 44,9,9±7,4 kg/m^2^ en préopératoire à 32,51±3,7 kg/m^2^ un an après la chirurgie, soit une perte moyenne de 63,35±6,5% de leur excès de poids. Selon les critères de Reinhold, cette perte pondérale était satisfaisante [14].

Ce travail rapporte les résultats d'une activité chirurgicale encore peu fréquente en Afrique noire, la chirurgie bariatrique. Dans notre contexte, un excès pondéral est souvent associé à une réussite sociale et au bien-être, tandis que la minceur est un signe de maladie et de pauvreté [15]. L'obésité et le surpoids sont toutefois associés à une morbi-mortalité importante. Ainsi 44% des cas de diabète, 7% des infarctus du myocarde et 44% de certains cancers sont attribués au surpoids et à l'obésité [16]; de plus, 3-4 millions de décès dans le monde en 2010 étaient dus à une surcharge pondérale [17]. Dans notre étude, tous les patients avaient au moins une comorbidité liée à l'obésité. La chirurgie bariatrique occupe une place importante dans la prise en charge de ces patients, car elle demeure la méthode la plus efficiente de traitement de l'obésité morbide et de ses complications [18]. L'augmentation rapide de la prévalence de l'obésité en Afrique [19,20] nécessite donc le développement d'une offre de chirurgie bariatrique; c'est dans cette optique que cette pratique a débuté dans notre service en 2016.

Notre étude a retrouvé, durant la première année suivant la chirurgie, une perte pondérale constante et régulière chez nos patients. Ce résultat est corroboré par les données de la littérature sur la SG, avec un %PEP à moyen terme variant entre 57 et 80% [21-23]. La SG, même dans un environnement à ressources limitées, entraîne une perte pondérale conséquente chez les patients. Cet effet sur la perte pondérale s'explique par la diminution importante du volume de l'estomac après une SG, les 2/3 de l'estomac étant emportés durant la procédure. Une autre explication réside dans la diminution de la sécrétion de ghréline, hormone responsable de la sensation de famine et sécrétée par le fundus (qui est sectionné durant la SG); la sensation de famine est ainsi diminuée chez les patients [24,25]. Cette action est d'autant plus importante que la SG crée un système à haute pression entre un pylore conservé et une lumière gastrique diminuée, entrainant de ce fait une sensation rapide de satiété chez le patient au décours d'un repas. L'effet de la conservation ou non de l'antre gastrique sur la perte pondérale restant sujet à controverse [12,26-28], nous préférons dans notre technique, débuter la section gastrique à 4 cm du pylore, diminuant ainsi le risque de sténose.

Les facteurs prédictifs d'un succès à long terme sur la perte pondérale après chirurgie bariatrique sont: un âge du patient inférieur à 40 ans, un IMC inférieur à 50 kg/m^2^, une équipe expérimentée pour la conduite de la procédure, une reprise de l'activité physique après l'intervention et un changement de comportement alimentaire [29]. Nous pensons que la stagnation de la perte pondérale, observée chez nos patients après les 12 mois suivants la chirurgie, pourrait s'expliquer par une mauvaise observance des deux derniers facteurs suscités. Un suivi rigoureux des patients après ce délai par l'équipe de diététique pourrait réduire cette stagnation pondérale.

Si quelques auteurs africains ont proposé la réalisation de procédures de chirurgie bariatrique par voie ouverte dans le but d'en faciliter l'accessibilité à nos patients souvent démunis [30,31], l'abord coelioscopique demeure l'abord standard pour la réalisation de ces interventions en raison de ses nombreux avantages. Notre temps opératoire moyen de 192 min était supérieur à celui rapporté dans la littérature qui est de 90 min [32]. Cette différence pourrait s'expliquer par la courbe d'apprentissage, la SG étant assez récente dans notre pratique. Si certaines équipes réalisent déjà la SG en ambulatoire [33], la durée moyenne d'hospitalisation de nos patients était de 4,7 jours. En début d'expérience et hanté par le risque de survenue d'une fistule gastrique, nous préférons à date de ne pas réaliser cette procédure en ambulatoire.

Nous pensons que les résultats satisfaisants sur la perte pondérale rapportés dans ce travail peuvent être obtenus par toute équipe africaine entrainée à la réalisation de cette procédure en particulier et à la prise en charge chirurgicale de l'obésité en général. Par ailleurs, nous avons déjà démontré la faible morbidité et la mortalité nulle de la SG dans notre contexte [34]. La pratique de la SG devrait donc être encouragée dans le strict respect de ses indications. La réalisation de SG nécessite toutefois une bonne maitrise de la coelio-chirurgie; une jeune équipe africaine de coelio-chirurgiens ne devrait pas débuter leur pratique par cette procédure.

Les limites de ce travail sont liées à son caractère monocentrique et à l'effectif limité de notre échantillon (21 patients), nous obligeant à rester modeste dans toute généralisation de nos résultats. Le caractère rétrospectif de la collecte des données ne semble pas avoir eu une influence péjorative sur l'étude car tous les patients opérés ont pu être colligés, preuve de la bonne tenue des dossiers de chirurgie bariatrique dans notre service. Notre étude n'a pas investigué l'évolution postopératoire des comorbidités chez nos patients. Il a toutefois été démontré que la chirurgie bariatrique entrainait une rémission, voire une guérison, de certaines pathologies telles que le diabète, l'apnée du sommeil, l'hyperlipidémie et l'hypertension artérielle [22]; d'où le concept de chirurgie métabolique [35]. La principale force de ce travail réside sur l'évaluation de la perte pondérale un an après la chirurgie bariatrique.

## Conclusion

La SG, bien que relativement nouvelle dans notre contexte à ressources limitées, donne des résultats satisfaisants sur la perte pondérale à moyen terme, soit une perte moyenne de 63,5% de l'excès de poids un an après la chirurgie. Sa pratique doit être encouragée, dans le strict respect des indications de chirurgie bariatrique.

### Etat des connaissances sur le sujet

- L'obésité morbide est une réalité en Afrique sub-saharienne;
- La « sleeve gastrectomy » est la technique de chirurgie bariatrique la plus usitée dans le monde;
- La perte de l'excès de poids un an après une sleeve gastrectomy varie entre 57 et 80%.

### Contribution de notre étude à la connaissance

- Un an après la « sleeve gastrectomy » à Yaoundé (Cameroun), les patients perdent en moyenne près des 2/3 de leur excès de poids;
- La perte pondérale un an après une « sleeve gastrectomy » à Yaoundé (Cameroun) est satisfaisante selon les critères de Reinhold;
- Un an après la « sleeve gastrectomy », la perte pondérale des patients opérés à Yaoundé (Cameroun) stagne.

## Remerciements

Les auteurs tiennent à remercier monsieur Samuel Dimbeng et monsieur Ambassa, infirmiers dans notre service, pour leur précieuse aide durant la réalisation de ces procédures chirurgicales et la conservation des dossiers de nos patients. Nous remercions également le docteur Guy Wafeu, statisticien, pour son appui dans l'analyse des données de ce travail.
